# Radiation exposure of eyes, thyroid gland and hands in orthopaedic staff: a systematic review

**DOI:** 10.1186/2047-783X-17-28

**Published:** 2012-10-30

**Authors:** Chandrasekharan Nair Kesavachandran, Frank Haamann, Albert Nienhaus

**Affiliations:** 1 Centre for Epidemiology and Health Services Research in the Nursing Profession (CV care), University Medical Centre Hamburg-Eppendorf, Martinistraße 52, Hamburg, 20246, Germany; 2 Department of Occupational Health Research, Institute for Statutory Accident Insurance and Prevention in the Health and Welfare Services, Pappelallee 33/35/37, Hamburg, 22089, Germany; 3 Epidemiology Division, CSIR-Indian Institute of Toxicology Research, Lucknow, UP, India

**Keywords:** Radiation, Dose, Orthopaedic

## Abstract

**Background:**

Various procedures, especially minimal invasive techniques using fluoroscopy, pose a risk of radiation exposure to orthopaedic staff. Anatomical sites such as the eyes, thyroid glands and hands are more vulnerable to radiation considering the limited use of personal protective devices in the workplace. The objective of the study is to assess the annual mean cumulative and per procedure radiation dose received at anatomical locations like eyes, thyroid glands and hands in orthopaedic staff using systematic review.

**Methods:**

The review of literature was conducted using systematic search of the database sources like PUBMED and EMBASE using appropriate keywords. The eligibility criteria and the data extraction of literature were based on study design (cohort or cross-sectional study), study population (orthopaedic surgeons or their assistants), exposure (doses of workplace radiation exposure at hands/fingers, eye/forehead, neck/thyroid), language (German and English). The literature search was conducted using a PRISMA checklist and flow chart.

**Results:**

Forty-two articles were found eligible and included for the review. The results show that radiation doses for the anatomical locations of eye, thyroid gland and hands were lower than the dose levels recommended. But there is a considerable variation of radiation dose received at all three anatomical locations mainly due to different situations including procedures (open and minimally invasive), work experience (junior and senior surgeons),distance from the primary and secondary radiation, and use of personal protective equipments (PPEs). The surgeons receive higher radiation dose during minimally invasive procedures compared to open procedures. Junior surgeons are at higher risk of radiation exposure compared to seniors. PPEs play a significant role in reduction of radiation dose.

**Conclusions:**

Although the current radiation precautions appear to be adequate based on the low dose radiation, more in-depth studies are required on the variations of radiation dose in orthopaedic staff, at different anatomical locations and situations.

## Review

### Background

Orthopaedic surgeons and staff are exposed to ionising radiation during a variety of procedures. In general, orthopaedic staff are exposed to both direct and scattered radiation during procedures. Over the past few decades, orthopaedic surgery procedures using fluoroscopy screening has increased [1-5]. Reports indicate that among the procedures that require fluoroscopic monitoring, closed locked femoral nailing is responsible for a high level of scattered radiation exposure among primary surgeons [3,6,7]. According to recent reports, improvements in image intensification technology have led to a reduction in the fluoroscopic time required for similar procedures [8-10]. Since the introduction of mini C-arm devices, fluoroscopic imaging is now routinely used in treating fractures in the emergency room and for outpatient and surgical orthopaedic procedures [5].

The cavalier use of fluoroscopic equipment by orthopaedic surgeons (for example, direct handling of the tube, placing hands directly in the field during operation of the machine) clearly breaches radiation safety guidelines [3,11]. Biplanar fluoroscopy, or the use of one fluoroscopic unit in two planes, is necessary during certain procedures. This is to monitor and prevent the leakage of polymethylmethacrylate outside the confines of the vertebrae and often requires the use of significant amount of ionising radiation [12].

The International Commission on Radiological Protection [13] has established the standards for radiation protection including the dosage limits. The maximum annual permissible upper dose limit is 20 mSv for the body, 150 mSv for thyroid or eyes, and 500 mSv for hands (International guidelines, ICRP). However, the dose limit for non-classified staff (for example, orthopaedic surgeons) is only 30% of these limits (that is, 150 mSv for hands) [14]. The recommended occupational dose of radiation for medical staff in Germany is 500 mSv for hands, 150 mSv for eyes and 300 mSv for thyroid [15].

The secondary (scattered radiation) dose distribution around the patient is non-uniform and does not exactly follow the inverse law as in the case of a point source and that a large number of dosimeters should be worn by personnel to record the dose absorbed by various body parts like eyes/forehead, neck/thyroid and fingers or hands. Multiple site measurement (not achievable on a routine basis) is expensive and uncomfortable for the practitioner, but permits a reasonable estimation of the spatial dose distribution [16]. The use of single dosimeters may lead to an underestimation of the effective dose. But, if the dosimeter is worn outside the lead apron it overestimates the effective dose. The inappropriate or slack use of protective devices and/or bad practice (for example, placing hands in the direct X-ray beam) could result in high doses in unexpected sites and poor correlation of dosimetric data [16]. It is suggested that dosimeters should be located under the lead apron (for whole body estimation), outside the apron at shoulder level, on the thyroid protector and at the hand [17,18].

In many situations, the effective whole body radiation dose is only a fraction of the dose to a single organ or tissue [19-21]. In such cases, the individual organs become the critical factors in the assessment of radiation hazards [22]. Orthopaedic surgeons face a greater risk of radiation exposure to hand [3,9,14,19,20,23] than radiologists and cardiologists [24,25]. Most of the studies on radiation exposure to healthcare workers (HCWs) are confined to whole body exposure. The source of radiation may come directly from the primary beam or indirectly from scattered radiation [14]. The hands, eyes and thyroid glands are more susceptible to radiation exposure among medical staff due to their proximity to primary radiation. It was this concern that prompted us to assess mean cumulative annual and per procedure radiation dose received at hands, eyes and thyroid in orthopaedic staff during different situations including procedures, work experience, using PPEs, etc. using a systematic review.

### Methods

#### Search strategy and screening

**Figure 1:**
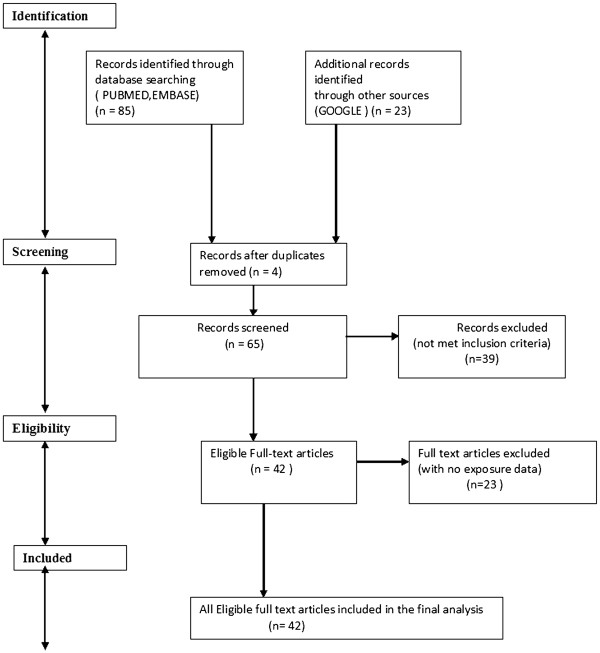
Identification, screening, eligibility and inclusion of data sources for the study.

The literature database was extracted by a systematic search of the PubMed and EMBASE databases and search engine (Google) using appropriate keywords: ‘radiation dose’ combined with ‘dose’, ‘orthopaedic’, ‘surgeons’, ‘technical’, ‘nurses’, ‘hands’, ‘fingers’, ‘neck’, ‘thyroid’, ‘eyes’, ‘forehead’ and ‘radiation safety’ for the period 1980 to 2011. We also searched through the reference lists of the selected studies and included appropriate publications in our work. Identification, screening, eligibility and inclusion of database for the study are shown in a flow chart (Figure 1). The literature search was conducted using a PRISMA flow chart [26] and the review protocol for PRISMA was based on the website information at http://www.prisma-statement.org/statement.htm.

The inclusion of publications for the present study was based on the following criteria:

Study design: Cohort, cross-sectional study, case–control study

Study population: Orthopaedic surgeons or their assistants

Exposure: Doses of workplace radiation exposure at different anatomical locations (hands/fingers, eye/forehead, neck/thyroid)

Language: German and English

### Study quality

The methodological quality of the studies was assessed as ‘moderate’ or ‘good’. A study was deemed to be of ‘moderate’ quality if it did not include dosimetric measurements of the eyes, thyroid gland and hands of orthopaedic staff. A study was rated as ‘good’, if the radiation dose of orthopaedic staff for these anatomical locations (eyes, thyroid and hands) was measured and included for analysis. Other studies related to radiation-related concerns in orthopaedic staff were also discussed in the study. All the authors of the present study carried out literature screening and quality evaluation independently. Their findings were then compared and in the event of disagreement, a consensus was reached by means of discussion. The risk of bias across studies and in individual studies was minimised using the study quality strategy. The synthesis of the study was done by plotting scatter plot graph based on the radiation dose in different studies for anatomical positions.

### Results

The study identified 85 records from databases and 23 records from other sources for the appropriate keywords (Figure 1). The records from databases were extracted from their home page of the website. Sixty-five records were screened based on the relevance of the topic and inclusion criteria. Forty-two articles were found eligible and included for the review. Twenty-three papers with no exposure data were considered to be of moderate quality and were excluded from the study (Figure 1).

**Figure 2:**
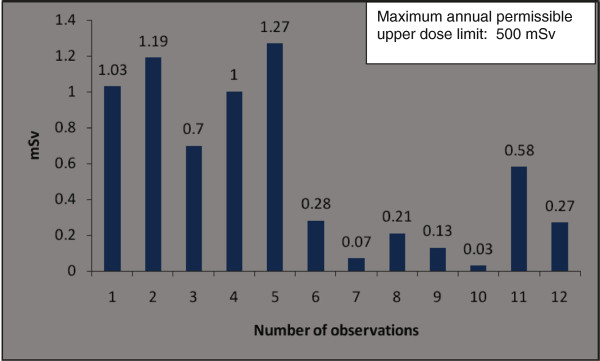
**The annual mean cumulative radiation dose received for hands/wrists among orthopaedic surgeons and technical assistants (observation number 1, 2).** Number of observation [reference]: 1 [9],2[9],3[14],4 [9],5 [9],6[3],7 [17],8[1],9 [6],10 [33],11 [11],12 [32].

**Figure 3:**
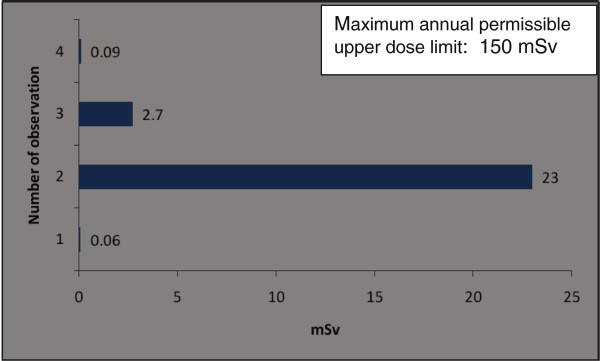
**The annual mean cumulative radiation dose received for eyes/forehead among orthopaedic surgeons and technical assistants (observation number 1).** Number of observation [reference]:1 [16],2 [1],3 [27],4 [32].

**Figure 4:**
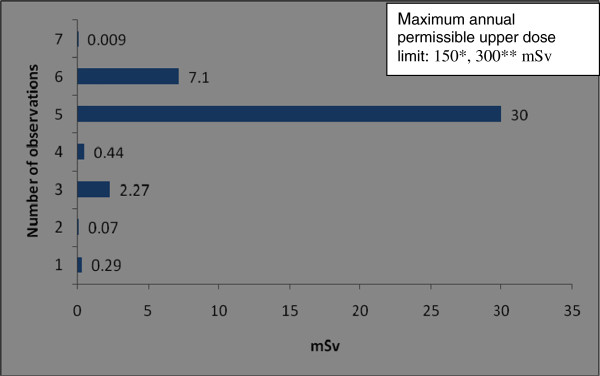
**Annual mean cumulative radiation dose received for thyroid among orthopaedic surgeons.** Number of observation [reference]: 1 [1],2 [6],3 [31],4 [30],5 [29],6 [27],7 [24]. *reference 14, ** reference 15.

**Figure 5:**
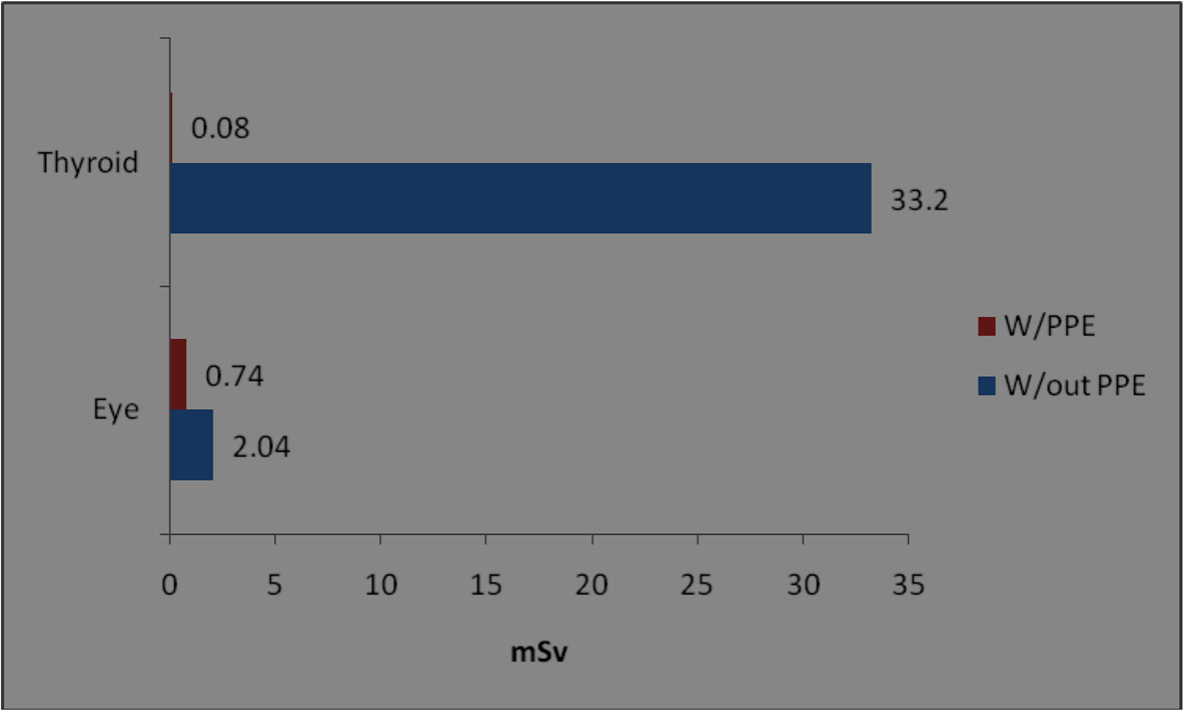
**Annual mean cumulative radiation dose received for hands, thyroid, eyes/forehead among orthopaedic surgeons during with and without personal protective equipment.** W/PPEs - with personal protective equipment; W/out PPEs-without personal protective equipment; eye [12]; thyroid [9].

**Figure 6:**
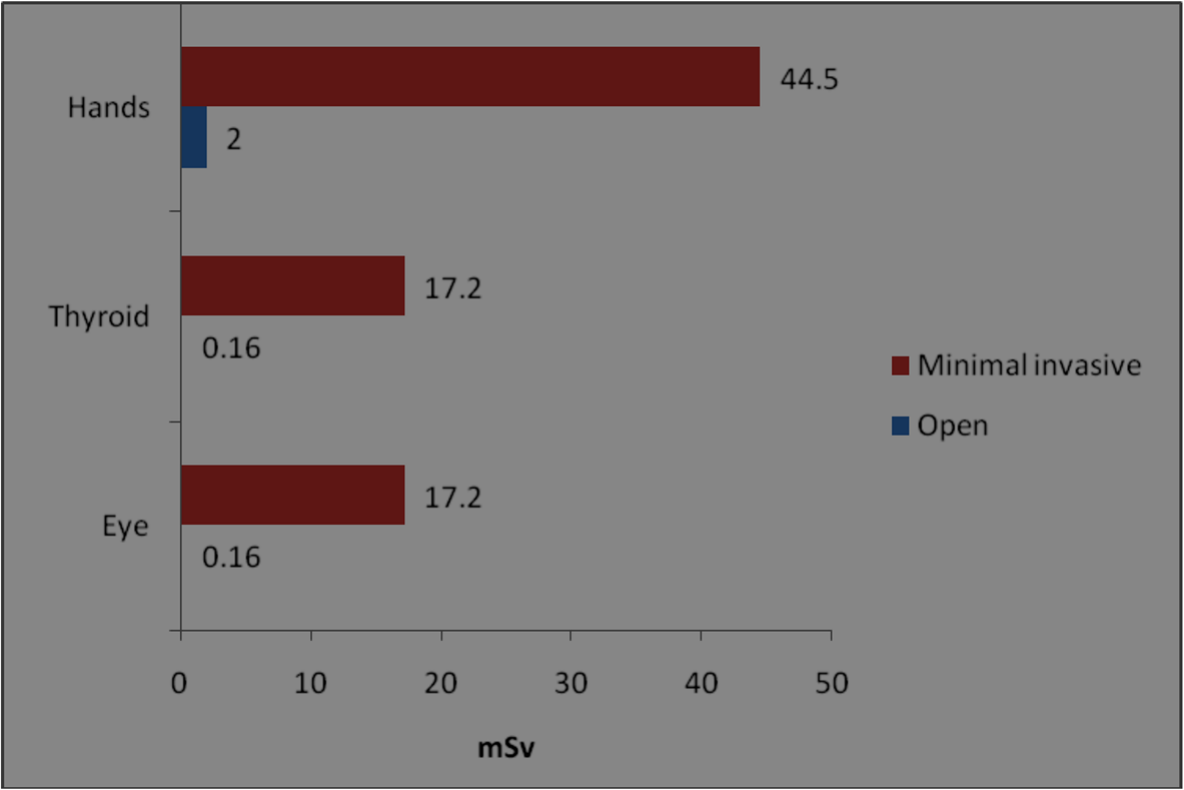
**Annual mean cumulative radiation dose received for hands, thyroid and eyes/forehead among orthopaedic surgeons during open and minimally invasive procedures.** The data shown in the figure were from an earlier study [28].

**Figure 7:**
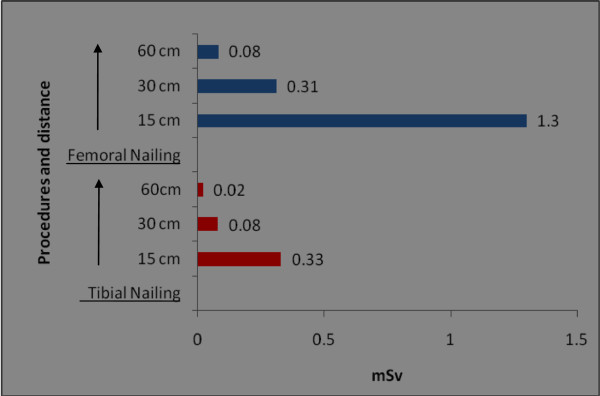
**The annual mean cumulative radiation dose received for hands/wrists among orthopaedic surgeons at femoral and tibial nailing procedure at different distances.** The data shown in the figure were from an earlier study [10].

**Figure 8:**
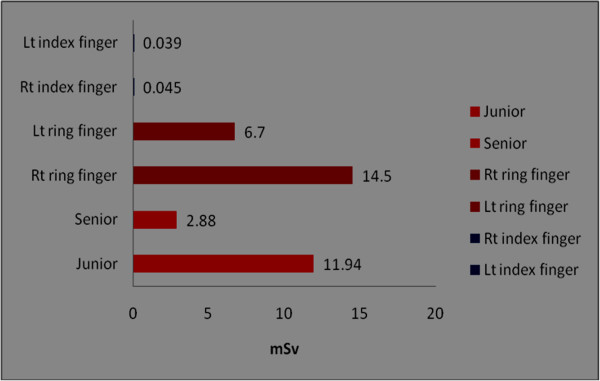
**The annual mean cumulative radiation dose received for hands/wrists among orthopaedic surgeon at different anatomical locations.** Left and right index finger [24], right and left ring finger [27], work experience (that is, senior and junior surgeons) [34].

**Figure 9:**
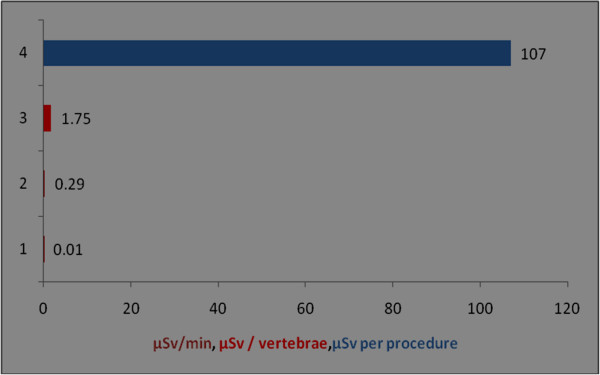
**Radiation dose received for hands/wrists among orthopaedic surgeons during per procedure.** Y axis represents the number of observations; observation number 1 is from technical assistant; different colours of bar denotes different units of observation; serial number of observation and references: 1 [16], 2 [16], 3 [40], 4 [27].

**Figure 10:**
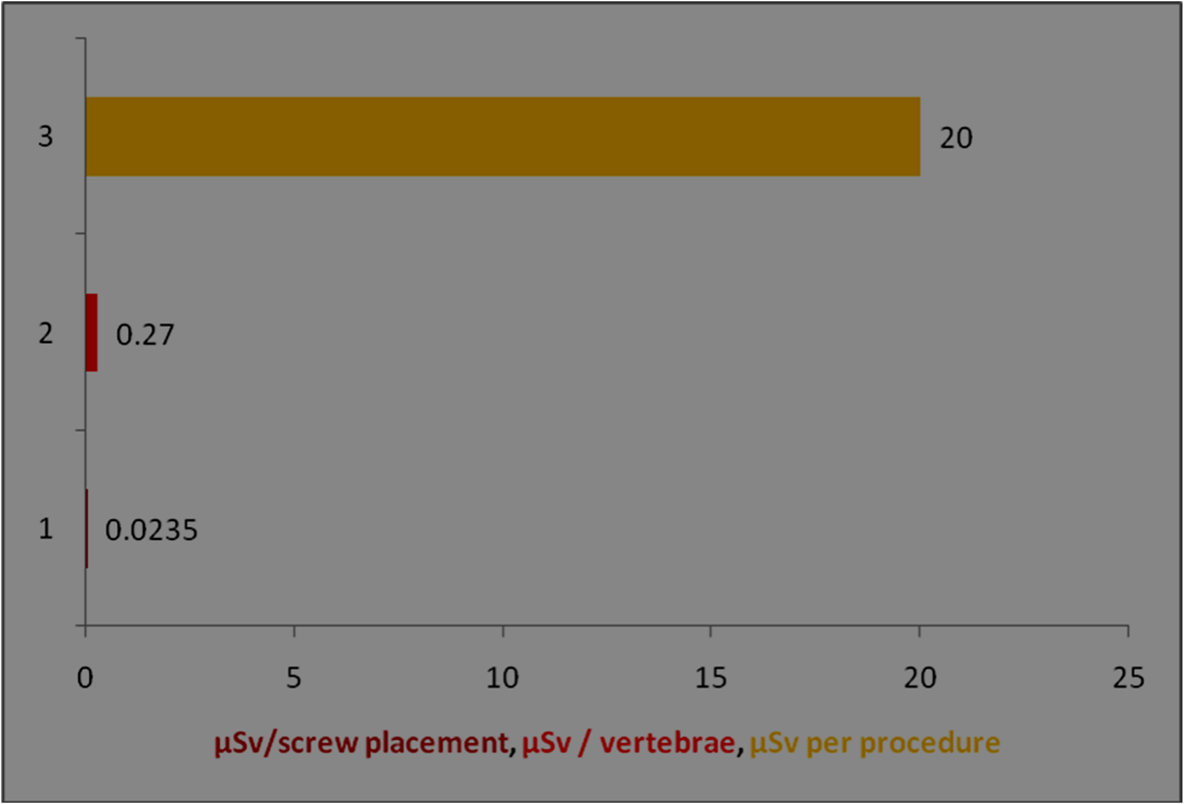
**Radiation dose received for eyes/forehead among orthopaedic surgeons during per procedure.** Y axis represents the number of observations; different colours of bar denotes different units of observation; serial number of observation and references: 1 [40], 2 [40], 3 [27].

The orthopaedic procedures in this study include fluoroscopic investigations for tibial nailing, femoral nailing, etc. A systematic review of the literature produced nine studies of the thyroid/neck region [1,6,9,22,27-31], five studies of the eyes/forehead [1,23,27,28,32], and 15 studies of hands/fingers [1,3,6,9-12,14,20,22,27,28,32-34]. There are studies with all three anatomical locations, some studies mention only two locations and others mention only one anatomical location. Hence references are repeated in different anatomical locations. Only observational studies based on mean of cumulative annual radiation dose were selected for analysis. Figure 2 shows that the observed annual radiation dose for hands/fingers of orthopaedic staff is low compared to the recommended limits, but with variations from 0.03 to 1.27 mSv. The observed annual radiation dose for eyes/forehead in orthopaedic staff is very low compared to the recommended limits, but with variations from 0.06 to 23 mSv (Figure 3). The doses of radiation measured at the thyroid gland/neck area of orthopaedic staff during surgical procedures were very low compared to the recommended dose, but with variations from 0.07 to 30 mSv (Figure 4). Personal protective equipments (PPEs) plays a very important role to reduce radiation which is shown in the Figure 5 for eyes and thyroid. There is a considerable difference between the mean cumulative radiation dose received for open and minimally invasive procedures; the radiation dose received for minimally invasive procedure among surgeons is higher compared to the open cases (Figure 6). Different procedures, namely femoral and tibial nailing, also show varied radiation dose response among surgeons at different distance (Figure 7). Procedures conducted at a short distance such as 15 cm show a higher radiation dose received among surgeons compared to a longer distance (60 cm). The anatomical locations (left and right index finger, left and right ring finger) and work experience (senior and junior surgeons) also play an important role in radiation dose received as shown in Figure 8. The right ring finger receives a higher radiation dose than left. Junior surgeons receive higher radiation doses than senior surgeons due to work experience. Even though recommended doses are not available for per procedure radiation dose received among orthopaedic staff, there is a considerable variation of dose received at hands/wrists (Figure 9) and eyes/for head (Figure 10).

The recommended annual dose limit for the extremities is 500 mSv. In the case of minimally invasive procedures, the highest measured dose to hands was only 44.5 mSv [28]. The radiation dose measured on left ring fingers (14.5 mSv) was higher than on right fingers (6.7 mSv) [28]. In another report [22] a minimal difference in radiation dose between the right (0.045 mSv) and left index fingers (0.039 mSv) was found. The staff using PPEs show 0.74 mSv compared to 2.04 mSv in cases without PPE.

An earlier report [10] on surgeons carrying out tibial nailing shows 0.33 mSv (at a 15-cm distance), 0.081 mSv (at a 30-cm distance), 0.021 mSv (at a 60-cm distance). For surgeons carrying out femoral nailing, the radiation dose reported was 1.272 mSv at 15 cm, 0.34 mSv at 30 cm and 0.080 mSv at 60 cm distance [10].

Higher radiation doses (33.2 mSv) were reported [9] at the thyroid region in orthopaedic surgeons who had not worn lead thyroid protection compared to those surgeons who had used lead thyroid protection (0.08 mSv). Thus the radiation dose among unprotected surgeons is 415 times higher than among surgeons who wore lead thyroid protection. According to a recent study, radiation doses at the thyroid/eyes received during open surgery were lower (0.16 mSv) compared to minimally invasive cases (17.2 mSv) [28].

### Discussion

Radiation exposure to the staff during a variety of orthopaedic procedures has been evaluated in earlier reports [1-4,6,9,11,12,14,22,23,27,28,30-42]. Among these studies, the studies pertaining to radiation exposure to the hands, eyes and thyroid, during fluoroscopically assisted procedures have demonstrated that the doses are well below the recommended levels of annual occupational radiation exposure. There is a wide variation among the radiation dose received at hands, eyes and thyroid glands which may be attributed to different types of orthopaedic surgery procedures, work experience, distance using PPEs, and so on. Even though studies on per procedure doses are available, no prescribed permissible limit is yet suggested for each procedure. Translating per procedure dose value into monthly or annual worker dose is difficult [43].

There is a high radiation doses were recorded from dominant index fingers and particularly the fingertip in orthopaedic surgeons [14]. The fingertip dose is high, while that of the finger base is below the acceptable limit. The high dose measured at the fingertip might be due to exposure to the primary beam during surgical protocols. Although surgeons and members of surgical teams are aware that their hands should never be exposed to primary radiation, it is not unusual for fingers to be accidentally caught in the beam [14]. In 15% of procedures it was reported that the surgeon’s hand had been caught in the fluoroscopy beam [19]. Most of the recorded doses occurred during brief exposure of the hands to the beam [9]. If the surgeon’s hand enters the primary beam, the dose is 100 times higher than the dose received at a distance of 15 cm from the beam, that is secondary (scattered) radiation [44]. The above observation shows that when the surgeons hand enters the primary beam (between the focus and the patient), there will be a large dose rate, whereas, when the surgeons hand is between patient and image intensifier, it will be a small dose rate.

Most of the studies indicate that the hands and fingers of orthopaedic surgeons receive the highest doses of radiation exposure [11,22,34,44,45] and this finding is independent of surgical skill [34]. The higher dose is of particular relevance in orthopaedic practice because of the proximity of the hands to the beam during radiation [22]. An earlier report suggests that a surgeon must perform 75 closed intramedullary (IM) femoral nailings with proximal and distal interlocking in 1 year to expose his or her hands to one-tenth of the maximum permissible dose [34]. Although it seems unlikely that an orthopaedic surgeon will reach the annual limit of radiation exposure of the hands, there is no threshold dose of radiation above which radiobiological effects (malignant cell transformation) could occur [34].

An increase in thyroid malignancy has also been reported among orthopaedic surgeons [19], but the study did not correlate the malignancy with radiation dose. The use of thyroid shielding and leaded glasses is often neglected by staff in the operating room [11]. This attitude could result in malignant conditions as reported earlier [19]. The radiograph position also plays a crucial role in radiation exposure to staff. If the radiograph tube is positioned above the patient, the exposure to the upper extremities, thyroid and eyes is four to five times higher [23,46].

The per procedure radiation exposure for eyes was 0.01 mSv/min [23] and 0.27 μSv/vertebrae [40] and 0.023 μSv per screw placement [41]. Assuming an average exposure of 40μGy per 5-min procedure, an unshielded surgeon operating approximately 20 cm from the mini C-arm beam could perform approximately 12,500 procedures before reaching established annual occupational eye limits, and 1,250 procedures before reaching established annual occupational whole body deep radiation limits [5].

Distance was established as one of the most important factors affecting radiation dose. The lateral fluoroscopic C-arm unit should be positioned so that the operator and medical staff are at a maximum distance from the patient surface where the primary radiograph beam enters the body [12]. An earlier report [47] offers insights into the relationship between radiation recorded in the operating room during interlocking intra-medullary nailing and the distance of the radiation monitor from the patient. During 7 min of fluoroscopy, the dose of radiation was 0.17 mSv at a distance of 40 cm, and 0.02 mSv at a distance of 80 cm. Increasing the distance from the source in order to decrease radiation exposure is a beneficial guideline for operative personnel. However, the dilemma for surgeons is that close proximity to the beam is routinely required for procedures like placement of interlocking screws. In addition, other dose reduction techniques based on geometric considerations, such as positioning the patient adjacent to the image intensifier, may be limited by the practical demands of the procedure [5]. The radiation dose received at left finger and hands are more than right hands or fingers [27]. This is due to the close proximity of the left side of the staff during the procedure.

Trainees and middle grade surgeons had a higher radiation exposure during proximal screening of long bones for identifying the insertion site of the guide pin. The middle grade surgeons experienced a significantly longer exposure time than consultants [10]. This difference in fluoroscopy time in relation to the experience of the surgeon has been reported previously [21]. Trainees were at higher risk while performing intramedullary nailing and whilst assisting surgeons. Thus the use of radiation is more consistent and standardised with a skilled surgeon [34]. However, even for fluoroscopically intensive musculoskeletal procedures such as IM nailing (for 4 to 5 min of fluoroscopy), the primary surgeon would have to perform several thousand procedures to reach the annual recommended limit [11].

The assistant surgeon, nurses and anaesthetists are better protected against radiation from the fluoroscopy compared to orthopaedic surgeons, thus their exposure is reduced to nearly immeasurable values. This is also due to staff rotations, which ensures that assistant surgeons and nurses do not assist all radiation procedures and hence their exposures are limited [22,23,48,49]. Earlier findings suggest that the levels of radiation dose observed in orthopaedic surgeons are unlikely to be associated with an increase in risk of congenital abnormalities or childhood malignancies in their children [50].

Although the current radiation precautions appear to be adequate based on the low dose radiation, there are still concerns regarding the safe practices followed among orthopaedic staff to reduce radiation dose ‘as low as reasonably achievable’ (ALARA). Some of them are discussed below.

### Awareness of radiation safety

In a recent report [51], it was observed that the surgical trainees in orthopaedic trauma surgery lack essential knowledge of ionising radiation and do not observe radiation safety principles. Lax use of fluoroscopic procedures by orthopaedic surgeons as reported earlier [3,11] may be due to a lack of awareness. Hence all orthopaedic staff should be made properly aware of radiation risks and receive radiation safety training. The staff may participate in an annual radiation awareness/training programme conducted by the radiation protection agency.

### Low dose exposure for a long time

Low dose of radiation was observed among the orthopaedic staff based on the available literature, which raises the question as to how low a dose must be if it is to be safe for each procedure? Staff who work for 20 to 30 years will probably have been exposed to low radiation for longer duration. There is uncertainty in predicting the effects of low dose radiation, hence it is wise to act on the basis that there is no safe dose of radiation [22].

### Distance and time

Distance is another limiting factor for radiation exposure as observed from the study for performing different orthopaedic procedures. The question is: what distance may be deemed safe for each of the procedures? This is a challenging task for the staff since in most cases they are very close to the radiation source for most of the procedures. Reduction of fluoroscopy time can also reduce the radiation dose, which is largely under the control of the surgical team [11].

### Dosimetry

A single dosimeter under a protective apron for whole body is not sufficient to measure radiation doses to other parts of the anatomy, for example eyes/head, fingers/hands, neck/thyroid glands. One dosimeter above apron and one below apron is a good practice in case of heavily irradiated personnel. Rarely, one additional in the hand (ring) is used. Modern dosimeters like thermo luminescent dosimeters (TLDs) on forehead, at thyroid gland, on lead collar, second and third finger, hands should be worn by staff during procedures to assess the exact radiation dose at these anatomical locations [11,22,27]. Staff prefers not to use TLDs as they feel it affects their skills and causes discomfort during various procedures.

### Personal protective radiation equipment

In particular, the use of thyroid shields as well as leaded eye protection is often neglected in the operating room [11]. Lead shields significantly reduce the effective exposure by several orders of magnitude [9,23], for example, reducing the dose level to the hands by as much as 33% [11]. Radiation attenuation gloves can offer an additional form of protection equipment during procedures. Without eye or hand protection, the total radiation exposure dose to these areas would exceed the occupational exposure limit after 300 cases per year [40]. Radiation reduction procedures and shielding devices such as a lead collar and goggles can lead to a significant decrease of mortality and morbidity of radiation sensitive tissue such as thyroid gland and eye lens [27].

The strength of the study is the systematic strategy followed for literature review with limited bias in the selection of relevant literature and analyse the global data on the radiation dose measurements in orthopaedic staff. The protective measures represented in the review suggest the need for strict implementation of regulatory guidelines for radiation safety among orthopaedic staff. The non-uniformity of the measured dose from different instruments (company), experience of staff and regulations in different countries may be a limitation for difference in the radiation doses among the staff. Studies are to be undertaken on radiation dose measurements among orthopaedic staff in Asia pacific region, where the number of procedures conducted annually are higher than the other continents.

## Conclusions

Although the current radiation precautions appear to be adequate based on the low dose radiation, more in-depth studies are required on the variations of radiation dose in orthopaedic staff, at different anatomical locations and situations.

## Abbreviations

ALARA: As low as reasonably achievable; HCW: Healthcare worker; ICRP: International commission on radiological protection; IM: Intra-medullary; μGy: Microgray; μSv: mGy/min, Milligray/minute/Microsievert; mSv: Millisievert; PPE: Personal protective equipment; PPRE: Personal protective radiation equipment; PRISMA: Preferred reporting items for systematic reviews and meta-analysis; TLD: Thermo luminescent dosimeters.

## Competing interests

All authors declare that they have no competing interests.

## Authors’ contributions

CNK and FH have made substantial contributions to the conception of the article and have been involved in revising the manuscript critically for important intellectual content. They have given final approval of the version to be published. AN has made substantial contributions to the conception and design of the review. He has been involved in drafting the manuscript and has given final approval of the version to be published.
